# A sustainable approach to recycling of polylactic acid with environmentally friendly reagents

**DOI:** 10.1016/j.susmat.2025.e01320

**Published:** 2025-04

**Authors:** Giacomo Damonte, Alberto Vallin, Leonardo Giribaldi, Alessandro Pellis, Minna Hakkarainen, Sathiyaraj Subramaniyan, Pietro Campaner, Orietta Monticelli

**Affiliations:** a Universitá degli Studi di Genova, Dipartimento di Chimica e Chimica Industriale, Via Dodecaneso 31, 16146 Genova, Italy; b KTH Royal Institute of Technology, Department of Fibre and Polymer Technology, Teknikringen 58, 100 44 Stockholm, Sweden; c AEP Polymers, Area Science Park Ed. Q1, Basovizza SS 14, km 163.5, 34149 Trieste, Italy

**Keywords:** PLA, Recycling, Alcoholysis, Biodegradable formulations, Zinc stearate, *Star*-shaped polymers

## Abstract

An important aspect before large-scale production and application of bioplastics such as polylactic acid (PLA), is the need to close the life cycle of the material to reduce the need for first-generation biomass and to prevent waste accumulation in the environment. In this work, starting from a high-mass linear commercial PLA, a depolymerization route based on a bulk alcoholysis reaction in the molten state was developed. For this purpose two polyalcohols, pentaerythritol and dipentaerythritol, and an environmentally friendly catalyst, i.e., zinc stearate, were utilized. The formation and specific polyalcohol dependent structure of star-shaped oligomers characterized by a low glass transition temperature was confirmed by spectroscopical and thermal analysis. Indeed, ^1^H NMR characterization evidenced that the most effective polyalcohol was pentaerythritol, which at the highest concentration in the reaction mixture, namely 10 wt.-%, allowed most of the hydroxyl groups to react, resulting in a system with a T_g_ of about 20 °C, which was much lower than that of the starting linear polymer, characterized by a T_g_ of about 60 °C. Moreover, GPC as well as DSC analysis in particular demonstrated the active role of zinc stearate in the transesterification reaction, as the samples prepared without adding the catalyst to the reaction mixture showed a modest reduction in Tg and molecular weight, which decreased from 92,000 g⋅mol^−1^ to 1700 g⋅mol^−1^ for the starting linear polymer and the resulting oligomer, respectively, in the case of the sample prepared with the highest amount of PE and with the addition of zinc stearate.The films produced from the star-shaped PLA oligomers were characterized by poor mechanical properties, but the high concentration of alcohol-functionalities could make them applicable in various polymer formulations. The *star*-shaped polymers were thereby blended with a multifunctional epoxide from renewable sources. The reactivity and compatibility of the two components was proved along with the specific role of zinc stearate remaining from the alcoholysis, in promoting the reaction between the two compounds. Indeed, the produced materials proved to be homogeneous, manageable and also completely enzymatically degradable.

## 1 Introduction

The end-of-life of polymers is increasingly at the focus of scientific and industrial interest, as raising the durability and circularity of polymer materials is in line with the requirements of sustainable economy [1]. Bioplastics respond to the need to limit the exploitation of fossil fuels, and many of them are susceptible to biodegradation e.g. industrial compost can be the targeted end-of-life [2,3]. Nevertheless, a wider use of such materials in many fields also requires an analysis of their potential recyclability [4]. The need to recycle bioplastics is also related to the fact that many of these materials are produced from renewable resources that are diverted from food use [5]. In addition, similar to traditional fossil-based plastics, the slow degradation rate of many bioplastics can lead to accumulation of large amounts of waste or even pollution of the environment [4,6,7]. As the production volume of PLA and other bioplastics is increasing, it is mandatory to also ensure their recyclability in closed or open loop [8]. Recycling of the PLA can be carried out by various methods, the simplest being the mechanical recycling [9–13]. Although the process is easily scalable, it suffers from degradation reactions, which are facilitated by moisture and high temperatures, a phenomenon that can only be reduced by the addition of antioxidants, anti-hydrolysis agents and chain extenders [14,15]. In particular, Badia et al. demonstrated that the performance of PLA decreases significantly after the second processing step [11]. For 3D printing process, it was reported that PLA can outlast two cycles before a significant drop in viscosity occurs, making the material unsuitable for further reprocessing [12]. A similar behavior was found by Brüster et al. for plasticized PLA, which was no longer suitable for the original application after multiple reprocessing cycles [13]. As the number of mechanical recycling cycles is limited and typically leads to downcycling, the development of chemical recycling could offer an attractive recycling option. However, despite recent academic and industrial interest, chemical recycling is still in its infancy [16–18]. Alcoholysis of PLA is proving to be one of the most promising chemical recycling methods, as it is both environmentally friendly and economically viable [8,19,20]. Indeed, the PLA alcoholysis products represent an excellent alternative to petroleum-based chemicals [21]. However, the main difficulties of PLA alcoholysis are the slow rate of alcoholysis and the low yield when only PLA reacts with the alcohol [19]. The reaction rate can however be significantly improved by using various catalysts [22–25]. Different from the past when the most commonly used catalysts were strong acids or bases [19], nowadays the catalysts used for the alcoholysis reaction of PLA mainly include organic catalysts, metal salts, and their complex catalysts, ionic liquids [22–25]. It is worth underlining that alcoholysis is a versatile process allowing the use of both monohydric alcohols and polyols, favoring the formation of products with star-shaped structures [26–30]. In the case of polyols, due to their sterical hindrance, the alcoholysis rate with PLA is very low compared with monohydric alcohols. As such, in order to promote the above reaction, it is necessary to add catalysts with relevant efficiency, such as tetrabutyl orthotitanate (TBT), which is one of the most applied catalysts. Nim et al. used the above catalyst by combining PLA with different kind of polyols [29,30], demonstrating that by increasing the mass ratio of PLA, the production of di-lactate, and poly-lactate also increased.

It is worth mentioning that the compounds obtained by PLA alcoholysis with polyols can be used to produce various materials such as polyurethanes, whose application areas are very wide, from packaging to materials with self-healing, biomedicine and coatings [31–33]. However, the further use of compounds derived from the alcoholysis of PLA generally requires intensive purification to remove solvents, catalysts and other components that could affect the final properties of the materials and their subsequent use [19]. Indeed, the above step is associated with a significant increase in the negative impacts and cost and thereby it sacrifies the scale-up potential of the entire process.

**Fig. 1:**
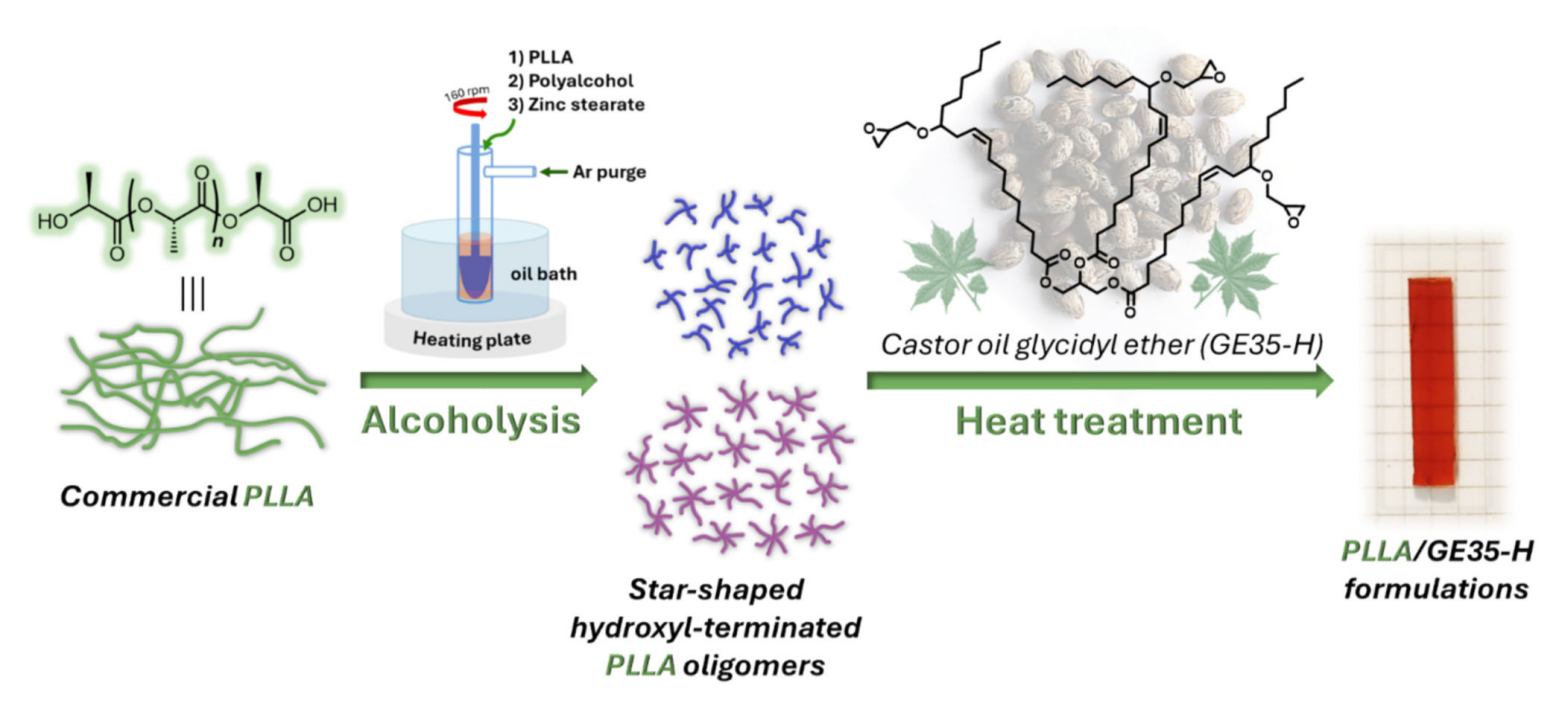
Scheme of the PLA alcoholysis reaction (polyalcohols = pentaerythritol or dipentaerhythritol) and the use of the resulting *star*-shaped polymers in the development of formulations based on castor oil triglycidyl ether (GE-35H).

It is clear that the application of PLA alcoholysis must take into account several aspects, such as: i) the use of polyalcohols from renewable sources, ii) the application of catalysts with low environmental impact and iii) the development of formulations that can be used without further purification. With this in mind, we investigated a method for PLA alcoholysis using potentially bio-based polyalcohols, namely pentaerythritol and dipentaerythritol, and environmentally friendly catalyst, namely zinc stearate, which is often applied in cosmetics [34] and in the polymer field as a lubricant [35,36] (Fig. 1). Zinc stearate, which is also used as a transfer catalyst for the saponification of fats [37], was not removed from the prepared systems and its specific role in the formation of formulations constituted by the developed *star*-shaped polymers from PLA alcoholysis and a commercial castor oil triglycidyl ether was investigated.

## 2 Experimental

### 2.1 Materials

A high molecular weight PLA, Luminy LX 175 (stereochemical purity: 96 % L-isomer, M_w_ = 245 kg/mol), was purchased from Corbion. *l*-lactide (L-LA) (purity >98 %) was kindly supplied by Purac Biochem (The Netherlands). Prior to polymerization, the monomer was purified three times by recrystallization in anhydrous toluene and dried under vacuum. Pentaerythritol (PE) (99 %), dipentaerythritol (DPE) (100 %), stannous octoate (Sn(Oct)_2_) (purity ≥96 %), dichloromethane (DCM) (stabilized with 0.002 % 2-methyl-2-butene), deuterated chloroform (CDCl_3_) (99.8 % atom D), tetrahydrofuran (THF) (≥99.9 %, anhydrous, inhibitor-free) and methanol (99.9 %) were purchased from Sigma-Aldrich® (Milan). Technical grade (Zn(St)_2_) was purchased from Faci S. p.A (Italy), ERISYS® Castor oil triglycidyl ether (referred to as GE-35H, EEW = 720 g/mol) was purchased from the Huntsman Advanced Materials (Belgium). All the reagents were used without further purification. *Humicola insolens* Cutinase (HiC) Novozym 51,032 was purchased from STREM chemicals.

### 2.2 Synthesis of star-shaped PLAs by alcoholysis in molten conditions

The star-shaped PLAs were prepared by alcoholysis from a high-molecular weight commercial PLA, by transesterification with two different polyalcohols (pentaerythritol, PE and dipentaerythritol, DPE) and Zn(St)_2_ as a catalyst. All the reactants were first dried for 72 h at 40 °C under vacuum. The PLA pellets were placed in the reactor, which had previously been heated to 195 °C. After the complete melting of the polymer, the calculated amount of polyalcohol (1 or 10 wt.-%) was added to the reactor and mixed into the molten PLA at 195 °C for 90 minwith mechanical stirring at 160 rpm. The catalyst (2 wt.-% based on the polymer) was then added and allowed to react for further 60 min. The reactions were carried out using a laboratory internal mixer with a mechanical stirrer, type RZR1 (Heidolph Instrument GmbH & Co, Schwabach, Germany), connected to an argon flow to maintain an inert atmosphere in the reactor. The obtained product was cooled to room temperature, removed from the reactor and dried at room temperature under vacuum. The same equipment, conditions and ratios were used for the reaction without catalyst (Table 1). The samples were defined by specifying the polyalcohol used (PE for pentaerythritol or DPE for dipentaerythritol), its percentage (1 or 10 wt.-%) and the presence or absence of the catalyst. For example PLA_PE_10_Zn refers to a sample prepared with 10 wt.-% of pentaerythritol and 2 wt.-% of Zn(St)_2_ as catalyst. To make a comparison with samples characterized by a controlled architecture, four- and six-arm *star*-shaped PLA were synthesized by ring-opening polymerization (ROP) according to the previously reported procedures [38,39]. In particular, Sn(Oct)_2_ was used as a catalyst and the amount of initiator was adjusted to obtain polymers of similar molecular weight as those produced by alcoholysis reaction.

### 2.3 Preparation of formulations based on star-shaped PLAs and castor oil triglycidyl ether (GE-35H)

For the preparation of the formulations, a specific amount of star-shaped polymer (PLA_PE_10_Zn or PLA_DPE_10_Zn), was blended with castor oil triglycidyl ether (GE-35H). Equimolar ratio between hydroxyl end group of star-shaped PLA and epoxy groups of the castor oil was used. The systems were mixed for 2 min at 140 °C using a metal spatula, to homogenize the mixtures. After this, the formulations were poured into a silicon mold (4 × 0.5 × 1 mm^3^) and treated at 150 °C for 48 h. Finally, the samples were removed from the mold and dried in a vacuum oven at room temperature for 24 h, before detailed characterization.

### 2.4 Characterizations

Nuclear magnetic resonance (^1^H NMR) spectroscopy analysis of the star-shaped PLAs were performed with a Jeol ECZ400R/S3 400 MHz using 10 mm NMR tubes and CDCl_3_ as a solvent. All the samples were dissolved in CDCl_3_ at a concentration of 15 mg/mL and analyzed at room temperature.

Fourier transform infrared (FT-IR) spectroscopy was carried out with Bruker “Vertex 70®” in ATR mode from 400 to 4000 cm^−1^.

Molecular weight characterization was performed on a HITACHI HPLC-GPC system in isocratic mode (0.5 mL/min flow rate; wavelength set to 280 nm), using 3 columns connected in series (Tosoh TSKgel Super H1000, Tosoh TSKgel Super H2000, Tosoh TSKgel Super H3000, 3 μm, 6 × 150 mm). The columns were thermostated at 40 °C and THF was used as mobile phase. 11 narrow molecular weight (M_W_) polystyrene standards were used for obtaining the calibration curve.

Differential scanning calorimetry (DSC) analysis of the samples was performed using a Mettler Toledo “DSC1 STARe System”. The samples were analyzed with a scanning rate of 10 °C/min in the temperature range from −50 to 200 °C and a nitrogen flow of 50 mL/min.

Thermogravimetric analysis (TGA) measurements were performed using a Mettler Toledo TGA instrument with a temperature program from 25 to 800 °C with a heating rate of 10 °C/min under a nitrogen flow of 50 mL/min.

The crosslinked fraction was determined by gel fraction (GF) and swelling ratio (SR) analyses using a good solvent for both star-shaped PLA and epoxidized castor oil. For this purpose, about 40 mg of the samples were weighed and immersed in 2 mL of DCM for 48 h. After that time, the samples were removed and dried, first at 25 °C at atmospheric pressure overnight, and then in a vacuum oven at 25 °C for 24 h. Finally, the samples were weighed, and GF were calculated using Eq. (1):

(1) $$GF[\text{\%}]=\frac{M_{d}}{M_{0}}•100$$

where M_d_ is the is weight of the dried samples and M_0_ is the initial weight. The SR was determined by weighing the crosslinked samples after 24 h kept in DCM and applying Eq. (2):

(2) $$SR[\text{\%}]=\frac{M_{wet}-M_{d}}{M_{d}}•100$$

where M_wet_ is the weight of the swollen sample.

Stress-strain elongation tests were performed at 25 °C by using an Instron Mechanical Tester (Instron 5565) (deformation speed = 0.5 mm/min, initial gage length l_0_ = 20 mm, pre-load = 0.05 N, *n* = 4) on 40x5x1 mm^3^ specimens that were previously in a desiccator over activated 4 Å molecular sieves.

Enzymatic degradation of the prepared materials was performed by placing single small rectangular specimens (dimensions = 5 × 2 × 2 mm^3^ ca., weight = 20 *±* 2 mg, n = 4) in 1.5 mL Eppendorf safe-lock tubes followed by 1 mL of a 5 μM solution of HiC in 0.1 M potassium phosphate buffer solution (KPO), pH = 8. Samples were incubated at 50 °C and weighed every 24 h until complete degradation was observed, changing the enzyme solution every 3 days. At the desired time point, the samples were removed from the enzyme solution, washed three times with an excess of milli-Q water, and then dried at 30 °C in a vacuum oven until constant weight before performing the gravimetric measurement of the weight loss using an analytical scale (*±*0.0001 g).

## 3 Results and discussion

### 3.1 Study of PLA alcoholysis in molten conditions

The alcoholysis of commercially available PLA in the molten state was performed using two types of polyalcohols, namely PE and DPE. Specifically, in this mechanism, one of the hydroxyl groups of the polyalcohols behaves like a nucleophile, attacking the carbonyl carbon of one of the ester groups of PLA. Any attack that occurs randomly along the PLA chains leads to their rupture, breaking the ester bond and forming two new PLA segments: one segment with a terminal hydroxyl group containing the alcohol group originating from the lactic acid repeating unit, and another consisting of a PLA chain ending with the new ester bond formed by the reaction between PLA and the polyol. Considering the random nature of the transesterification process in polyester alcoholysis, such attacks are expected to initially lead to the formation of linear structures (i.e. those formed when only one of the hydroxyl groups of the polyalcohol reacts with a PLA chain to form a PLA chain terminated with a polyalcohol monoester and a second chain that is “ejected” from the original PLA segment after reacting with the polyalcohol) [40]. By repeating this process several times, the degree of esterification of the hydroxyl groups of the polyalcohol can be gradually increased and the molecular weight of its PLA-bonded chains reduced at the same time. As already described in the literature, this process leads to the formation of a mixture of structures characterized by different degrees of substitution and chain lengths [29].

**Fig. 2:**
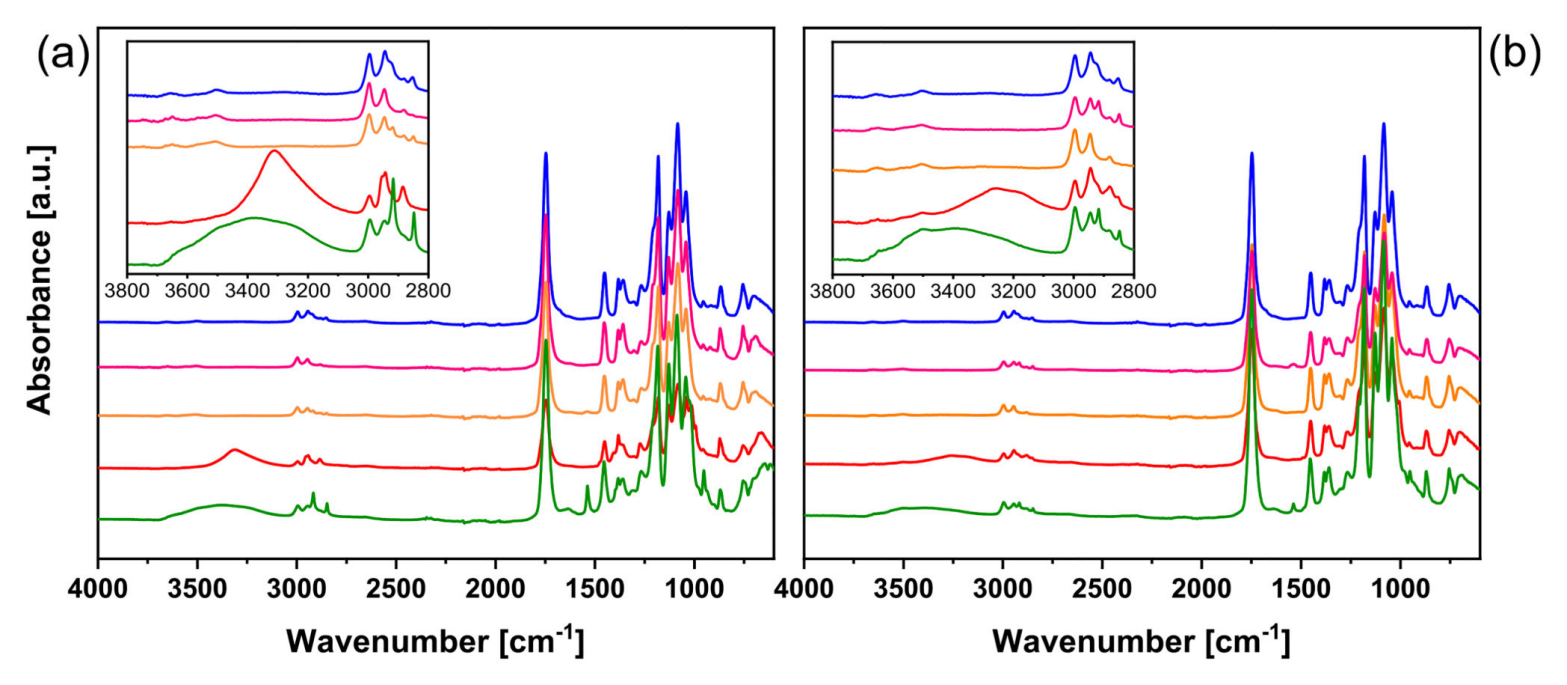
FT-IR spectra of samples prepared by alcoholysis with increasing concentration of PE (a) or DPE (b): neat PLA (blue), PLA_PE_1 or PLA_DPE_1 (pink), PLA_PE_1_Zn or PLA_DPE_1_Zn (orange), PLA_PE_10 or PLA_DPE_10 (red) and PLA_PE_10_Zn or PLA_DPE_10_Zn (green). The same colors are used for corresponding samples in both graphs.

The chemical transformations induced by the process were preliminarily investigated by FT-IR measurements, focusing on the effects of the polyalcohol concentration in the reaction mixture on the alcoholysis reaction using two clearly different amounts of PE and DPE (1 and 10 wt.-%). Moreover, to verify the role of the catalyst in the above reaction, samples treated for the same time and in the presence of the same polyalcohol but without Zn(St)_2_ were also examined. In Fig. 2, the spectrum of neat PLA is compared with the spectra of the samples treated with PE and DPE both in the presence and absence of the catalyst. The spectrum of PLA shows characteristic stretching frequencies for C=O, –CH_3_ asymmetric, –CH_3_ symmetric, and C-O, at 1746, 2995, 2946 and 1080 cm^*−*1^, respectively, while bending frequencies for –CH_3_ asymmetric and –CH_3_ symmetric are identified at 1452 and 1361 cm^*−*1^, respectively [41].

In all samples subjected to the alcoholysis process, the formation of a band between 3600 and 3100 cm^*−*1^ can be seen, the intensity of which increases with increasing the polyalcohol content in the reaction mixture. Although it proved difficult to detect differences between the samples prepared with the two polyalcohols, the comparison of the spectra shows that this band appears at higher wavenumbers in both the systems prepared with the catalyst compared with those without the catalyst, a phenomenon that suggests a different nature of the hydroxyl end groups. In order to better analyze and explain the described results, the samples prepared starting from the highest amount of PE, i.e., PLA_PE_10 and PLA_PE_10_Zn, were ground and soaked in water for 72 h, a solvent that can dissolve the unreacted polyalcohol. For the sample prepared without catalyst, PLA_PE_10, washing led to a significant decrease in the band between 3600 and 3100 cm^*−*1^ (Fig. S1), while this signal remained practically unchanged for the sample with catalyst, PLA_PE_10_Zn (Fig. S2). The data obtained by weighing the samples before and after washing also show that PLA_PE_10_Zn lost almost no weight, while PLA_PE_10 showed a loss of about 9 %. This corresponds closely to the 10 % of PE added, indicating that most of it remained unreacted in the system. These results, although preliminary, demonstrate the role of the catalyst in promoting the transesterification reaction, thus favoring the scission of the macromolecular chains with the formation of star-shaped structures. On this basis, it can be hypothesized that the polyalcohol reacts only minimally without the catalyst and remains dispersed in the polymer matrix.

**Fig. 3:**
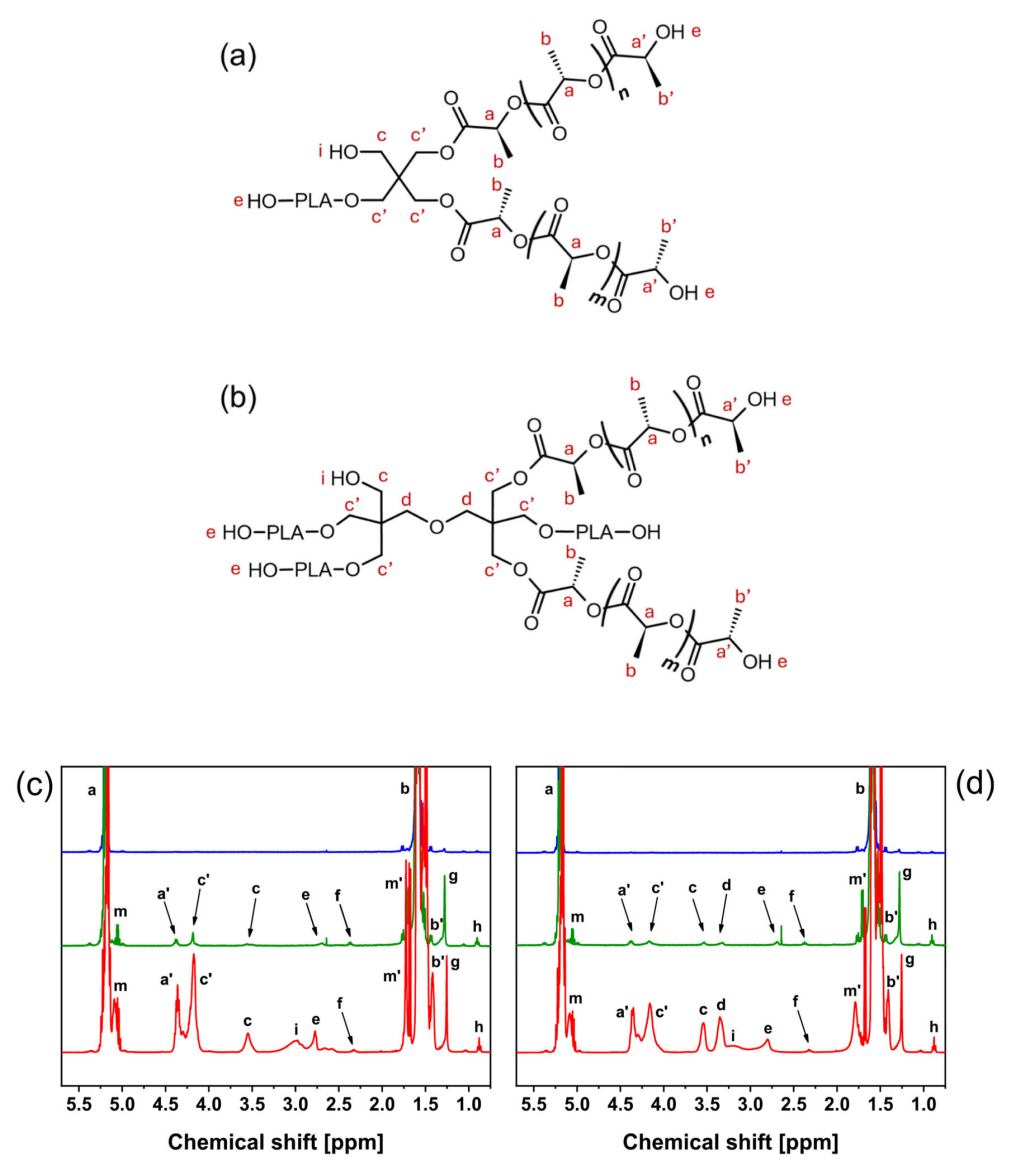
Examples of possible structures of star-shaped PLA oligomers obtained by alcoholysis reaction of PLA by using PE (a) or DPE (b). ^1^H NMR spectra of PLA and polymers prepared by alcoholysis with increasing concentration of PE (c) or DPE (d). PLA (blue), PLA_PE_1_Zn (green), PLA_PE_10_Zn (red). The same colors are used for corresponding samples in both graphs.

^1^H NMR spectroscopy was performed to elucidate in detail the chemical structure of linear high molecular weight PLA and the star-shaped oligomers prepared by ROP and alcoholysis, using PE or DPE as polyalcohols in both reactions. In detail, observing the spectrum of the linear high molecular weight PLA, shown in Fig. 3, two signals at 5.17 ppm (a, m, CH repetition unit PLA) and 1.59 ppm (b, m, CH_3_ repetition unit PLA) can be recognized, which are typical of the PLA backbone protons, as previously reported [42]. The absence of additional signals, related to the PLA chain end groups, typically present around 4.35 ppm and 2.74 ppm, can be ascribed to the small amount of end groups due to the high molecular weight.

The spectra of the *star*-shaped polymers prepared by ROP of l-lactide in the presence of PE and DPE as initiators, namely PLA_ROP_PE and PLA_ROP_DPE, are shown in Fig. S3. These spectra clearly exhibited the above signals at 4.35 ppm (a’, q, PLA-CH-OH) and 2.74 ppm (e, s, PLA-CH-OH) of PLA chain terminal protons, confirming the low molecular weights. This was further confirmed by GPC results. Moreover, peaks at 4.14 ppm in PLA_ROP_PE (c’, s, PE-CH_2_-O-PLA) and 4.13 ppm in PLA_ROP_DPE (c’, s, DPE-CH_2_-O-PLA) were detected, which can be attributed to the presence of the two different initiators. It is worth underlining that for all star-shaped PLA samples prepared by ROP, the ratio of the area between the methylene protons signal of the functionalized polyalcohol at 4.18 ppm and the methine proton signal of the PLA end groups at 4.35 ppm was close to 2. This indicates that ROP of lactide proceeds strictly on hydroxyl groups of the co-initiator molecules, resulting in polymers characterized by high chain-end fidelity.

In addition, two signals were found at 5.06 ppm (m, q, CH *l*-LA) and 1.70 ppm (m’, s, CH_3_ *l*-LA), which can be attributed to the presence of traces of *L*-lactide in these samples [43].

The sample prepared by alcoholysis showed very similar patterns to the oligomers synthesized by ROP. In detail, the spectrum of the sample prepared by alcoholysis with the addition of 1 wt.-% PE in the presence of Zn(St)_2_, namely PLA_PE_1_Zn, exhibited signals associated with PE and PLA chain ends at 4.35 ppm (a’, q, PLA-CH-OH), 4.18 ppm (c’, s, PECH_2_-O-PLA), 3.55 ppm (c, s, PE-CH_2_-OH), 2.74 ppm (e, s, PLA-CH-OH) and 1.42 ppm (b’, m, CH_3_ PLA chain terminal). The peaks at 2.32 ppm (f, t, CH_2_-COO stearate), 1.25 ppm (g, m, CH_2_ stearate chain), and 0.88 ppm (h, t, CH_3_ stearate chain terminal) confirmed the presence of Zn (St)_2_ in the material. Indeed, the signals related to the PLA chain end groups, which were not present in the starting polymer, indicate a reduction in the molecular weight of the sample as a result of the alcoholysis process. As expected, the spectrum of the sample with 10 wt.-% PE, PLA_PE_10_Zn, showed a significant increase in the intensity of the signals related to PE methylene protons and PLA chain end groups, indicating a further reduction in molecular weight after alcoholysis. Similar results were observed for samples prepared by alcoholysis of PLA with DPE at 1 and 10 wt.-%. Specifically, the sample prepared by adding 1 wt.-% DPE, namely PLA_DPE_1_Zn, showed similar signals to the PE-containing samples, but with slight shifts caused by the different DPE chemical structure, at 4.18 ppm (c’, s, DPE-CH_2_-O-PLA), 3.55 ppm (c, s, DPE-CH_2_-OH) and 3.34 ppm (d, s, CH_2_-O-CH_2_ DPE). Also in this case, the sample with 10 wt.-% DPE (PLA_DPE_10_Zn) showed an increase in the peak area at 4.35 ppm, relative to the terminal methines of the PLA chain, evidencing a further reduction in the molecular weight of the polymer compared to that of PLA treated with 1 wt.-% DPE. In addition, signals related to traces of lactide and the zinc-based catalyst were detected in all samples prepared by alcoholysis.

The signal around 3.55 ppm which, as previously reported, can be associated with the methylene protons of polyalcohols with free hydroxyl groups and which was present in all samples prepared by alcoholysis, deserves a more detailed comment. Indeed, the presence of the above peak demonstrates that the hydroxyl groups of polyalcohols cannot fully participate in the transesterification process with the ester bonds of PLA during the alcoholysis.

To better define this aspect, the degree of functionalization of the hydroxyl groups was indirectly determined for all samples, as reported in Table 2, by calculating the ratio between the signal area of the methylene units of the polyalcohols bound to the PLA chains and the sum of the areas of the methylene groups bound to both the free hydroxyl groups and the PLA chains, as shown in Eq. (3):

(3) $$\text{Reacted}\;\text{hydroxyl}\;\text{group}(\%)=\frac{A_{c^{\prime }}}{\left(A_{c}+A_{c^{\prime }}\right)}.100$$

where: *A*_*c*_′ = signal area of methylene units of polyalcohols bound to PLA chains, *A*_*c*_ = signal area of the methylene units of the polyalcohols bearing the hydroxyl groups.

As can be seen from the results in Table 2, the polymers prepared by ROP exhibited a higher degree of substitution of the polyalcohol hydroxyl groups by PLA chains, with PLA_ROP_PE and PLA_ROP_DPE showing almost complete functionalization, i.e., about 99 %. This finding shows no differences between the hydroxyl groups of PE and DPE in terms of effectiveness as co-initiators in the ROP, which can be attributed to the high catalytic activity of tin octanoate in promoting the polymerization reaction. As reported by Puchkov et al., under reaction conditions similar to those used in our work, this very active catalyst can promote the complete conversion of hydroxyl groups in a few minutes [44], which can be attributed to the fast initiation rate that prevents the preferential attack of monomer units on individual hydroxyl groups, leaving the reactivity of the remaining groups unaffected. In contrast, polymers prepared by alcoholysis of linear PLA with PE showed lower degrees of functionalization, namely 70 % and 83 % for PLA_PE_1_Zn and PLA_PE_10_Zn, respectively. The increase in the sample containing the highest PE concentration can be related to the higher polyalcohol content, which improves the reaction kinetics and functionalization also due to a reduced system viscosity, caused by the PE dissolution in molten PLA.

In the case of the DPE-based samples, the increase in polyalcohol concentration was found to modestly affect the percentage of functionalization, being 70 % and 72 % for PLA_DPE_1_Zn and PLA_DPE_10_Zn, respectively. In addition, it is worth noting that the value of the system prepared using the highest amount of polyalcohol is much lower than that of the corresponding PE-based sample, PLA_DPE_10_Zn. As reported by Puchkov et al., this difference could be related to a lower reactivity of DPE compared to PE, presumably due to the different steric hindrance caused by the highly branched structure [44]. These results suggest that the hydroxyl groups of the polyalcohol are not fully involved in the transesterification process with the PLA ester bonds during alcoholysis. Furthermore, a gradual decrease in the reactivity of these groups can be considered as their involvement in the transesterification reaction increases due to the steric hindrance generated by the increasing number of PLA chains around the polyalcohol core [45,46]. In other words, during alcoholysis the reactivity of the hydroxyl groups of the polyalcohols changes after the initial attack as they are bound close to a high molecular weight PLA chain. Nevertheless, it is important to underline that the reaction between hydroxyl groups of polyalcohols and PLA chains, even if partial, leads to significant reductions in the molecular weight of the samples, which is evidenced by the GPC results. Moreover, as with the samples prepared by ROP, the ratio between the area of the signal at 4.18 ppm and that at 4.35 ppm was close to 2, demonstrating that the newly formed hydroxyl PLA chain terminal groups are uniquely generated from the reaction of polyalcohol with PLA chains and not from other secondary undesired chain breaking processes. This also leads to the formation of oligomers characterized by high chain-end fidelity.

The influence of the catalyst on the alcoholysis reaction was further investigated by analyzing samples prepared without addition of catalyst to the reaction mixture. In particular, in the spectra of the samples prepared by adding different amounts of polyalcohols to PLA, but without catalyst, i.e., PLA_PE_1, PLA_PE_10, PLA_DPE_1 and PLA_DPE_10, shown in Fig. S4, no signals related to the methylene protons of the initiators bound to the PLA chains were observed, thus indicating that alcoholysis occurred only to a limited extent.

Further evidence of the importance of the catalyst addition is that the degree of functionalization of the polyalcohols in the samples prepared without the catalyst, i.e., PLA_PE_1, PLA_PE_10, and PLA_DPE_1 and PLA_DPE_10, was found to be zero (Table 2), demonstrating that the hydroxyl groups do not participate or react to any significant extent under these reaction conditions. These results confirm once again the utmost importance of the catalyst in promoting the transesterification reaction between the hydroxyl groups of polyalcohols and PLA ester bonds.

The change in molecular weight of the samples after the alcoholysis process compared to the neat PLA was evaluated by GPC analysis (Fig. S5). Table 3 shows the values of M_n_, M_w_ and dispersity (Ð) of PLA and the developed materials. The commercial PLA was characterized by high molecular weight with M_n_ around 90•000 g⋅mol^*−*1^. For the PE-based samples prepared without catalyst, a decrease in molecular weight was observed, with M_n_ of 30•000 and 24•000 g⋅mol^*−*1^ for the samples prepared from 1 % and 10 % polylalcohol, respectively. The same trend was observed when DPE was used, but the variation in molecular weight compared to the neat polymer was less than for the previously described samples: M_n_ was around 48•000 and 40•000 g⋅mol^*−*1^ for PLA_DPE_1 and PLA_DPE_10, respectively. These results underline the spontaneity of the alcoholysis process, which, however, seemed to be less influenced by the amount compared with the type of polyalcohol used. In contrast to this, the addition of the catalyst led to a considerable reduction in molecular weight, which was particularly significant for the systems prepared by adding 10 wt.-% polyalcohols as M_n_ was reduced to 1•700 and 5•100 g⋅mol^*−*1^ for PLA_PE_10_Zn and 5•100 for PLA_DPE_10_Zn, respectively. These results supported the NMR findings, proving that in the absence of the catalyst only a negligible part of the polyalcohol participated in the transesterification reaction and the increasing steric hindrance of the polyalcohol further decreased the reactivity of the hydroxyl groups, as shown by the fact that PE-based samples achieved a lower molecular weight than those prepared from DPE.

The thermal properties of the prepared samples were compared with those of the starting PLA using DSC measurements. The results are summarized in Table 3, while the corresponding curves are shown in Fig. S6. The DSC traces related to the second heating show a glass transition temperature (T_g_) of 60 °C for neat PLA, which also appeared to be completely amorphous. This is consistent with the behavior of this polymer described in the literature, where is reported to be characterized by slow crystallization kinetics promoted only by an annealing process [47]. For the PE-based samples, T_g_ of the polymers prepared without the catalyst decreased slightly to 57 and 55 °C for PLA_PE_1 and PLA_PE_10, respectively. More significant reductions in the glass transition temperatures occurred in the samples that were subjected to alcoholysis in the presence of the catalyst. In this case, the T_g_ of the sample prepared by adding the largest amount of PE to the reaction mixture, PLA_PE_10_Zn, decreased to 21 °C, which is about 40 °C lower than the T_g_ of the neat polymer. This result can be related to the decrease in the molecular weight of the above systems, which, as the GPC measurements demonstrated, was more significant in the case of the samples based on the use of the catalyst. It is worth underlining that in contrast to PLA_PE_1_Zn and PLA_PE_10_Zn, the two samples prepared without the addition of the catalyst showed a cold crystallization peak characterized by a similar enthalpy to the melting event, which proves the amorphous character of the samples. This difference can be explained by the fact that alcoholysis favored by the catalyst not only leads to the formation of structures with lower molecular weight, but also promotes the reaction of the hydroxyl ends of the polyalcohol, as demonstrated by NMR, facilitating the formation of star-shaped structures, characterized by a low tendency to crystallize. Indeed, it is possible to hypothesize that the fraction of the polyalcohol that reacts without catalyst leads to the formation of more linear structures, since the possibility that all the end functionalities of the polyalcohol reacts is limited, a phenomenon that can favor the crystallization of the shortened chains, at a temperature higher than the T_g_. Similar trends were also observed in the systems prepared with DPE, but the T_g_ of PLA_DPE_1_Zn and PLA_DPE_10_Zn were higher than those of the two homologs PLA_PE_1_Zn and PLA_PE_10_Zn, since the alcoholysis of the former leads to the formation of structures with lower molecular weight.

### 3.2 Development of formulations based on star-shaped PLAs and castor oil triglycidyl ether (GE-35H)

To verify a possible application of the *star*-shaped PLA-based polymers produced by the alcoholysis reaction, formulations with a commercially available multiepoxide, i.e., castor oil triglycidyl ether (GE-35H) were prepared. Indeed, the latter compound can be easily synthesized from castor oil, an inedible oil and a renewable resource for many chemical industries, mainly produced in Asia and Africa [48,49]. Unlike other vegetable oils, castor oil can be directly transformed into glycidyl ether by treatment with epichlorohydrin [50]. In general, castor oil-derivates were mainly applied as additives in the formulation of thermosets, e.g. based on bisphenol A [50], polyurethane [51] and epoxy acrylate [52].

Regarding the development of our formulations, it is worth underlining that, unlike other systems reported in the literature, purification of the alcoholysis-derived polymers was omitted to make the preparation of the material more easily scalable [19]. Films based on star-shaped PLAs, which, as previously reported, were characterized by a significant decrease in molecular weight compared to the starting polymer, exhibited poor mechanical properties. Indeed, the results of the mechanical tests highlighted the extreme fragility of the films produced by compression molding, a phenomenon related both to the low molecular weight and to the *star*-shaped architecture of the polymers, which limit the formation of entanglements between the macromolecular chains. However, the high functionality of the star-shaped PLAs potentially gives them high reactivity. On this basis, the combination of the hydroxyl-terminated PLAs with a multiepoxide was chosen, as their functional groups are potentially able to react and thus promote the formation of a homogeneous system [53]. To this end, the two components, the star-shaped PLA and the multiepoxide, GE-35H, were used in an equimolar ratio between the epoxide and hydroxyl functional groups, selecting the polymers with the lowest molecular weight, namely PLA_PE_10_Zn and PLA_DPE_10_Zn. The temperature used, 150 °C, was chosen to significantly reduce the viscosity to favor the intimate mixing of the two components (Fig. S7). For the reaction time, studies were taken into account in which the multiepoxide applied was combined with other systems for the production of thermoset materials [54]. It is worth underlining that the formulations prepared were homogeneous and transparent (Fig. S8), a result that provides a preliminary indication of the good miscibility between the two phases.

**Fig. 4:**
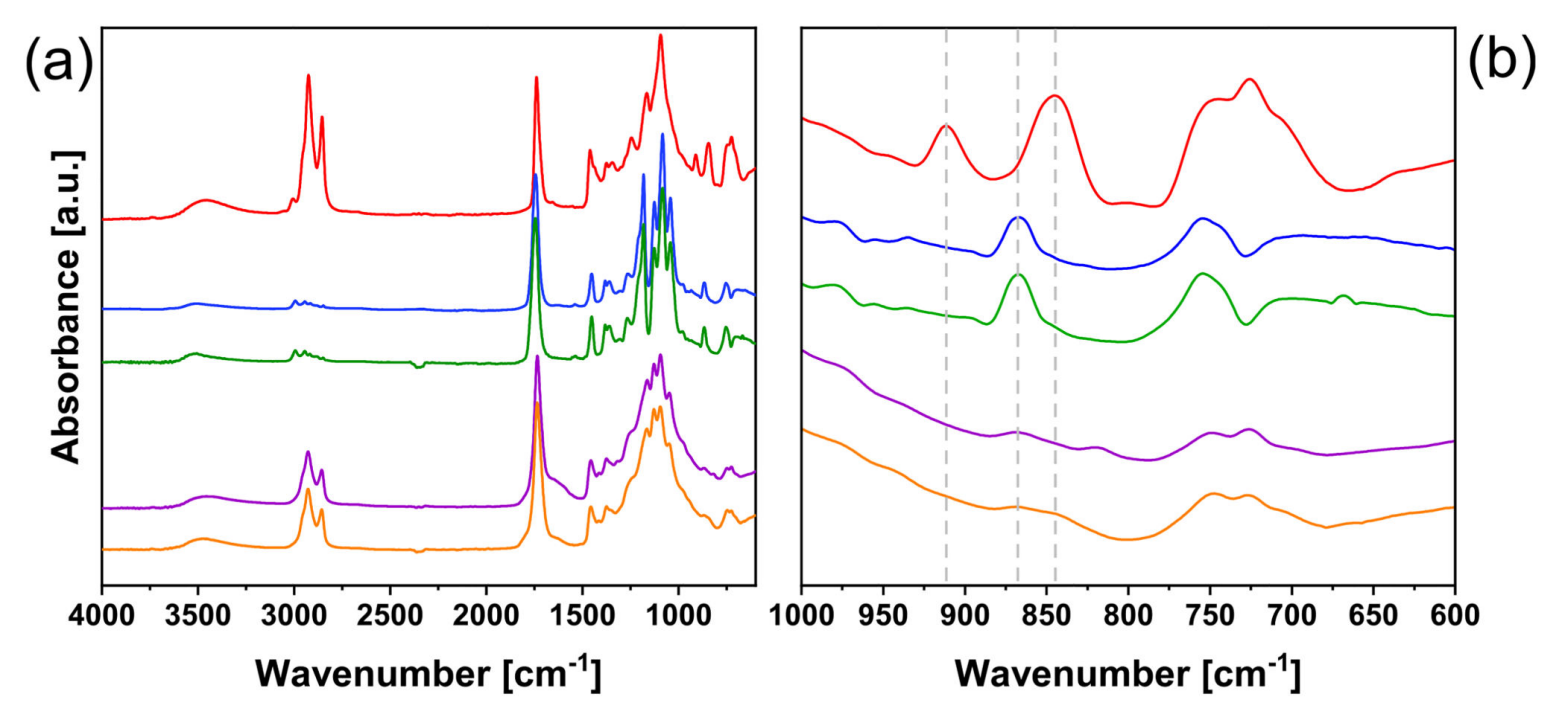
FT-IR spectra of: GE-35H (red), PLA_PE10_Zn (light blue), PLA_DPE_10_Zn (green), PLA_PE_10_Zn/GE-35H (violet) and PLA_DPE_10_Zn/GE-35H (orange). (a) from 4000 cm^*−*1^ to 800 cm^*−*1^ and (b) from 1000 cm^*−*1^ to 600 cm^*−*1^.

The FT-IR spectra of the two components mixed before heat treatment, reported in Fig. 4, show the characteristic bands of PLA [55] and the multiepoxide [56]. In the case of GE-35H, the bands at 911 cm^*−*1^ for the asymmetric ring deformation and at 845 cm^*−*1^ for the symmetric ring deformation of epoxy rings are clearly visible [56]. The heat treatment of the two components led to a significant decrease in the intensity of the two bands mentioned above, which highlights the reduction in epoxy functionalities due to their reaction with PLA end groups. It is worth underlining that it is more difficult to determine the disappearance of the hydroxyl functionalities, since the opening of the epoxide ring and its reaction with the end functionalities of PLA involves the formation of -OH groups.

To highlight the specific effect of the catalyst on the reactivity of the system, the multiepoxide was also combined with a polymer prepared by ROP using PE as initiator (PLA_ROP_PE) and characterized by the same molecular weight as that from alcoholysis, under the same conditions as the previously described samples. In particular, two samples were prepared, one by adding an amount of catalyst to the mixture corresponding to that contained in the polymers from alcoholysis (PLA_ROP_PE/GE-35H/Zn) and the other without (PLA_ROP_PE/GE-35H). The purpose was to evaluate the influence of catalyst, which was difficult to completely eliminate from the *star*-shaped polymers derived from the alcoholysis process. The decrease in the characteristic bands of the epoxy groups is clearly observed in both samples, demonstrating the spontaneity of the reaction at high temperatures (Fig. S9a). However, significant differences were observed by washing the materials with a solvent capable of dissolving both the multiepoxide and PLA, i.e., toluene. In the formulations heat-treated in the presence of a catalyst, the extracted fraction is significantly lower compared with the system without catalyst. The extracted amounts were 36 % and 53 % for the PLA_ROP_PE/GE-35H/Zn and PLA_ROP_PE/GE-35H samples, respectively. Moreover, it is relevant to underline that washing did not eliminate completely the polymer fraction in the mixture, as the FT-IR spectrum showed the characteristic peaks of the *star*-shaped polymer, particularly at 867 cm^*−*1^, even in the systems that underwent the washing treatment (Fig. S9b). On this basis, the results show that although the reaction was spontaneous, the presence of the catalyst remaining from the alcoholysis did not limit the subsequent reaction but rather accelerated the reactivity between the formulation components.

**Fig. 5:**
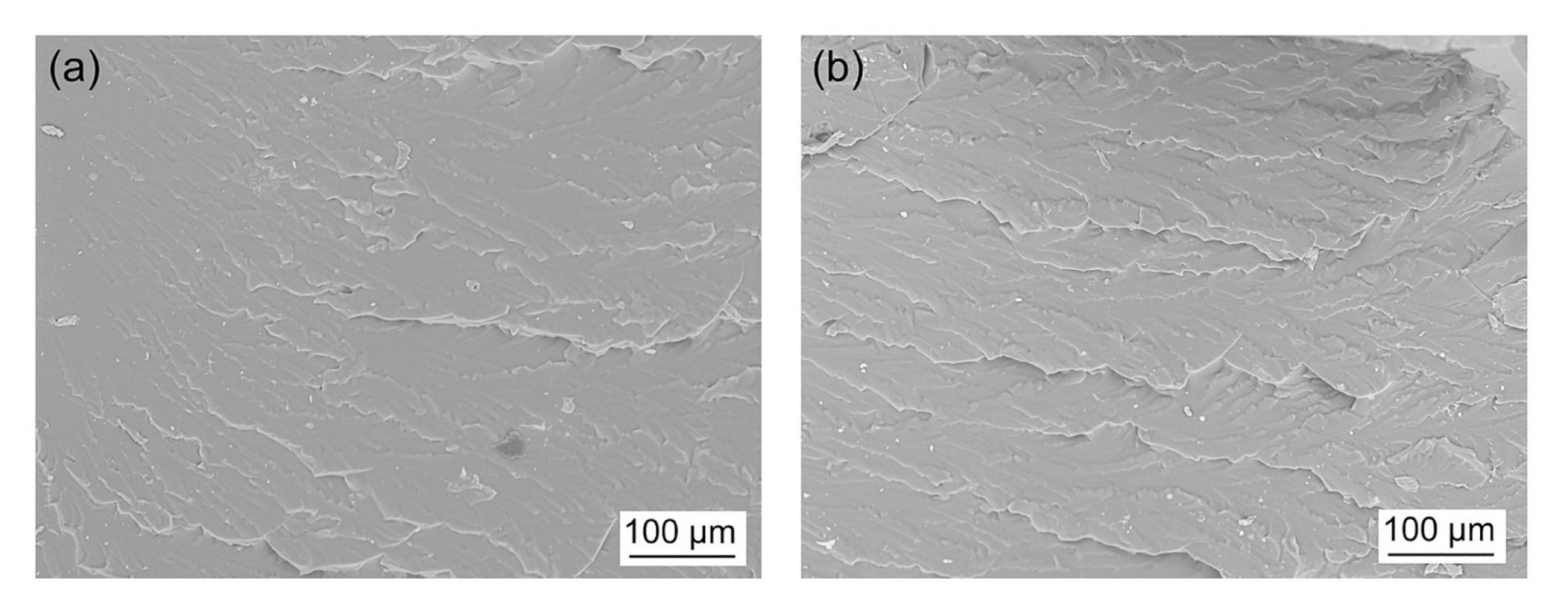
FE-SEM of: (a) PLA_PE_10_Zn/GE-35H and (b) PLA_DPE_10_Zn/GE-35H.

The morphology of the prepared materials was analyzed in detail by FE-SEM measurements, showing, as in the macroscopic analysis, a significant structural homogeneity, i.e., the developed samples did not show phase separation (Fig. 5).

The thermal properties of the formulations were analyzed by DSC measurements, comparing their thermograms with those of the starting materials (Table 4 and Fig. S10). Considering the second heating, both samples showed a glass transition temperature (T_g_) between that of epoxy and that of star-shaped PLAs. Indeed, T_g_ for PLA_PE_10_Zn/GE-35H and PLA_DPE_10_Zn/GE-35H was *−*9 °C and −25 °C, respectively. The difference in T_g_ between the two samples was in perfect agreement with the amount of epoxy used in the formulation, since to maintain a stoichiometric ratio between the -OH and epoxy groups, the amount of GE-35H used was higher in the case of the system based on PLA_DPE_10_Zn than that based on PLA_PE_10_Zn and consequently T_g_ of the former turned out to be lower. This result, together with the morphology, proves a good miscibility between the two compounds, and it is possible to hypothesize that the reactivity further facilitates this. The thermal degradation of the formulations was also compared with that of the starting compounds; the results of the TGA measurements are shown in Table 4. The comparison highlights that both the initial degradation temperature (T_onset_) and the temperature of the maximum degradation rate (T_max_) of the two formulations were higher than those of the *star*-shaped polymers from alcoholysis. T_onset_ values were 319 °C and 337 °C and T_max_ values 379 °C and 393 °C for PLA_PE_10_Zn/GE35H and PLA_DPE_10_Zn/GE-35H, respectively, while T_onset_ of 252 *°*C and 248 *°*C and T_max_ of 313 °C and 309 °C were recorded for PLA_-PE_10_Zn and PLA_DPE_10_Zn, respectively. This underlines that the formulation led to materials characterized by a high stability exceeding that of the starting systems. In addition, the fact that the degradation process was characterized by only one event again indicates chemical reactions taking place between the components.

Tensile tests were carried out to verify the applicability of the developed materials (Fig. **S11**). As previously reported, films based on star-shaped PLA exhibited poor mechanical properties and the samples were so fragile that they could not be tested, while the heat-treated materials were tested without experimental difficulties. The data obtained from the mechanical tests are summarized in Table 4, where Young’s modulus (E), tensile strength (σ_max_) and elongation at break (ε_break_) are given. Although the moduli obtained are limited at around 250 KPa, the samples show sufficient toughness to be handled. To explain this behavior, the fact that the starting epoxy is normally used as a plasticizing component in formulations based on high-strength resins must be taken into account. Moreover, it is worth underlining that the elongation at break values, which were similar for both systems, were around 11 %. These values are higher than those of the starting PLA, which, as widely reported in the literature, appears to be extremely fragile and is characterized by an elongation of 4 % [57]. The results therefore demonstrate the possibility of using the bio-based multi-epoxide not only as an additive but also as a main component in formulations, highlighting its potentially wider applications. In addition, specific applications are conceivable that do not require high mechanical properties but could exploit the functionality of the material due to the presence of the *star*-shaped polymers, e.g. in environmental remediation.

**Fig. 6:**
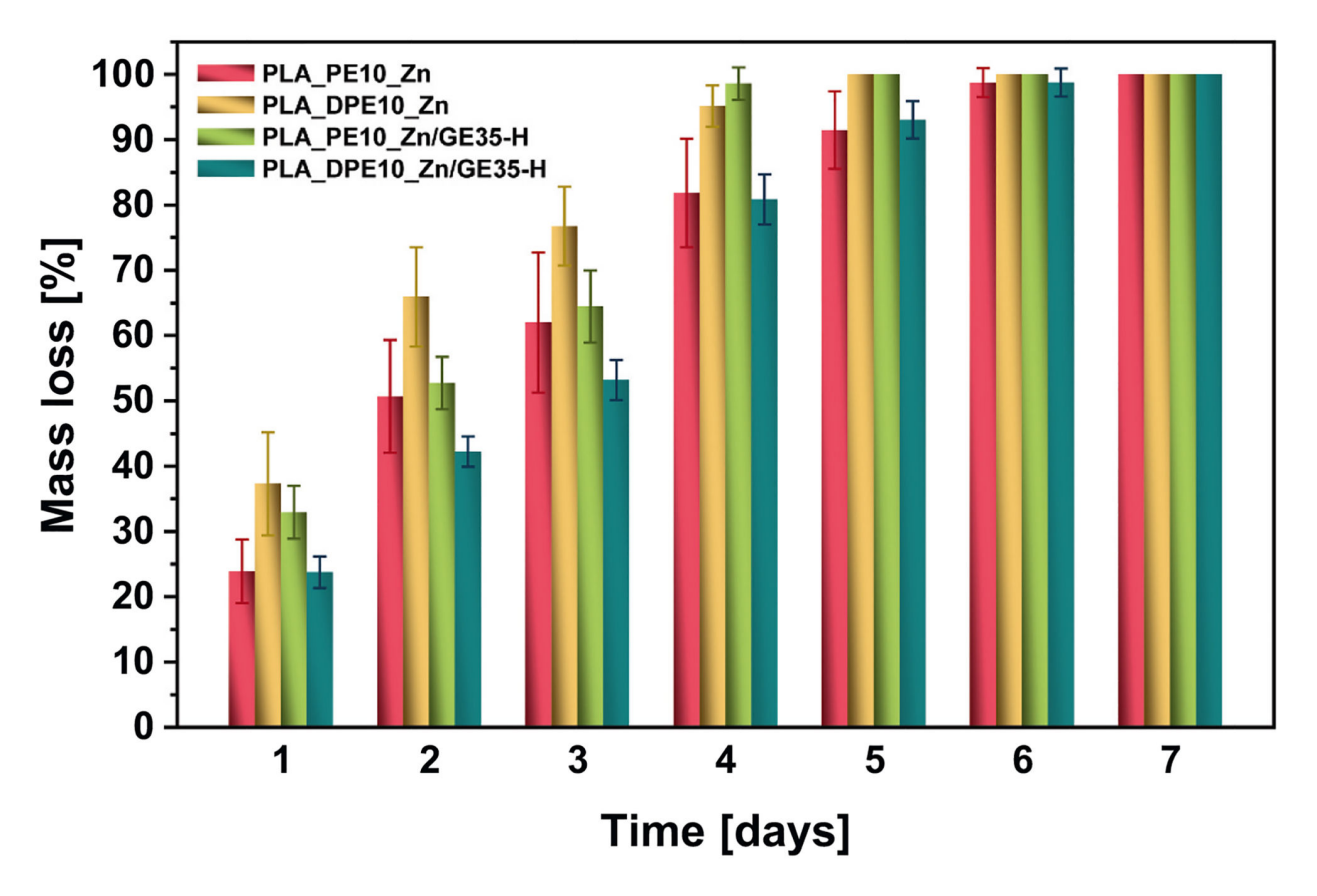
Enzymatic hydrolytic degradation profile of: PLA_PE_10_Zn, PLA_DPE_10_Zn, PLA_PE_10_Zn/GE-35H and PLA_DPE_10_Zn/GE-35H in a 5 μM cutinase solution in 0.1 M KPO buffer pH = 8, *T =* 50 ° C.

The enzymatic degradation of the star-shaped PLA from alcoholysis, PLA_PE_10_Zn and PLA_DPE_10_Zn, and their corresponding formulations, PLA_PE_10_Zn/GE-35H and PLA_DPE_10_Zn/GE-35H, was evaluated in the presence of HiC, a highly active hydrolytic enzyme belonging to the serine hydrolase family [58]. The above enzyme proved to be very effective in the degradation of polyesters [59]. The tests were carried out at 50 °C and pH = 8, conditions that accelerate the process, and the degradation was followed for 7 days, i.e., until the specimens were completely dissolved. Fig. 6 compares the mass loss of the neat polymers with that of their formulations. It was found that the star-shaped PLA produced by alcoholysis was completely degraded in seven days. Additionally, LC-MS analysis of the supernatant recovered after 3 days of enzymatic hydrolysis, in negative ion mode, confirmed the presence of species derived from PLA chains hydrolysis. In particular, for PLA_DPE_10_Zn (Fig. S12), lactic acid (LA, 3.28 min, *m*/*z*: 89.0), lactic acid dimer (2LA, 10.25 min, m/z: 161.2) and lactic acid trimer (3LA, 13.33 min, m/z: 233.2) were found. A direct comparison with the results reported in the literature is difficult, as linear high molecular weight polymers are generally considered. For example, Damonte et al. observed that dense PLLA films (40 μm thickness), based on a high molecular weight polymer, prepared by solvent casting and treated under very similar conditions (HiC 5 μm, 50 °C, PBS buffer 0.01 M, pH = 7.4) [60], showed a mass loss of about 20 % after 7 days of immersion in the enzyme solution. Similar results were reported by Huang et al. who investigated the degradation of hot-pressed PLLA films (100 μm thickness) with HiC and observed a mass loss of about 10 % after the same degradation time [61]. In this case, the final concentration of the enzyme was not explicitly stated, but only the enzymatic solution activity (2.0 mL of HiC 100 U/mL in phosphate buffer 0.1 M, pH = 7.5). The different behavior of our materials compared to the systems described above highlights that the reduction in molecular weight due to the alcoholysis process, combined with the formation of star-shaped structures, characterized by much lower glass transition temperatures and a higher free volume than the linear high molecular weight polymer, significantly facilitates the enzymatic degradation. Indeed, these findings also show the possibility of reducing the degradation time of the virgin polymer by applying a simple alcoholysis step, which could be particularly useful for the development of materials characterized by enhanced biodegradability.

Considering the behavior of our developed materials, it is extremely interesting to note that there are no significant differences in degradation rate between the neat polymers and the formulations and that all the materials were completely degraded to water-soluble products in seven days (Fig. 6 and Fig. S13). This result can be attributed to the fact that the attack of the enzyme on the polyester promotes the degradation of the entire system, thus favoring the dissolution of the films. The analysis of the supernatant from PLA_DPE_10_Zn/GE-35H (Fig. S14) showed that the hydrolytic process led to the formation of lactic acid (LA, 3.33 min, *m*/*z*: 89.2) and other unidentified compounds. In particular, these compounds are hypothesized to be fatty acids formed by the hydrolytic cleavage of GE-35H ester bonds. Moreover, the reaction between GE-35H and the star-shaped PLAs (e.g. in PLA_DPE_10_Zn/GE-35H) could lead to the formation of several additional products by forming ether (non-hydrolyzable) and ester (hydrolyzable) bonds through ring opening of the epoxide or transesterification of the ester moieties of GE-35H. Indeed, the increased chemical complexity resulting from the addition of GE35-H to the PLA matrix complicates the identification of these compounds, which are presumably formed during the hydrolysis of this fraction. Nevertheless, the enzymatic hydrolysis of both samples revealed the formation of lactic acid, a monomer that can be effectively separated from the mixture and recycled to produce PLA. These promising results point to the possibility of establishing a recycling loop for these systems, promoting their reuse and extending their life cycle in favor of their environmental impact.

## 4 Conclusions

Considering the need to design post-consumer pathways retaining the material value of PLA, an efficient and easily scalable recycling method characterized by a low environmental impact was developed. The approach, which is applicable to linear PLA recycling under solvent-free conditions, is an alcoholysis process that involves the use of potentially bio-based compounds, namely pentaerythritol (PE) and dipentaerythritol (DPE) as polyalcohols and zinc stearate as a catalyst, which has never been used for this type of reprocessing before. The properties of the *star*-shaped PLAs obtained depended on the type and concentration of polyalcohols, and they were characterized by low T_g_ and fast enzymatic degradability compared to the starting PLA. In particular, PE turned out to be the most efficient polyalcohol to promote PLA alcoholysis, leading to star-shaped PLA with a molecular weight of 1700 g⋅mol^*−*1^ and a T_g_ of about 20 °C when 10 wt.-% PE was added to the reaction mixture. The development of formulations prepared by a simple thermal treatment of star-shaped PLA from alcoholysis and a multifunctional epoxy from renewable sources, demonstrated both the potential application of the prepared systems and the favorable impact of the catalyst remaining from the alcoholysis process, which promoted the reaction between the formulation components, facilitating their compatibilization and enhancing the material’s final properties. The direct applicability of the formed star-shaped PLAs without need of a purification step is a significant advantage and step forward from common chemical recycling approaches were intensive purification with solvents clearly limits the sustainability and large-scale development. Films produced from the developed formulations are characterized by a higher strength than those prepared from the star-shaped PLA, while retaining the fast enzymatic degradability. These properties, combined with the simple and scalable production, make the developed recycling approach interesting from both a scientific and an application point of view.

## Supplementary Material

ESI

## Figures and Tables

**Table 1:** Reaction conditions used for alcoholysis and ring-opening polymerization (ROP) reaction.

Sample code	Catalyst	PE [wt.-%]	DPE [wt.-%]
PLA		−	−
PLA_PE_1	−	1	−
PLA_PE_10	−	10	−
PLA_DPE_1	−	−	1
PLA_DPE_10	−	−	10
PLA_Zn	Zn(St)_2_	−	−
PLA_PE_1_Zn	Zn(St)_2_	1	−
PLA_PE_10_Zn	Zn(St)_2_	10	−
PLA_DPE_1_Zn	Zn(St)_2_	−	1
PLA_DPE_10_Zn	Zn(St)_2_	−	10
PLA_ROP_PE	Sn(Oct)_2_	3.2	−
PLA_ROP_DPE	Sn(Oct)_2_	−	4.1

PE: pentaerythritol; DPE: dipentaerythritol

**Table 2:** Amount of reacted and unreacted hydroxyl functional groups in PLA samples prepared using different synthetical methods.

Sample	Reaction type	Polyalcohol type	Zn stearate [%]	Reacted hydroxyl groups* [%]	Number of reacted OH groups per polyalcohol molecule
PLA_ROP_PE	ROP	PE	0	99	3.96
PLA_PE_1	alcoholysis	PE	0	0	0
PLA_PE_10	alcoholysis	PE	0	0	0
PLA_PE_1_Zn	alcoholysis	PE	2	70	2.80
PLA_PE_10_Zn	alcoholysis	PE	2	83	3.32
PLA_ROP_DPE	ROP	DPE	0	99	5.94
PLA_DPE_1	alcoholysis	DPE	0	0	0
PLA_DPE_10	alcoholysis	DPE	0	0	0
PLA_DPE_1_Zn	alcoholysis	DPE	2	70	4.20
PLA_DPE_10_Zn	alcoholysis	DPE	2	72	4.32

* determined by ^1^H NMR using Eq. (3).

** determined by comparing signal intensity between X-CH_2_-O-PLA (where X = PE or DPE) and PLA CH-OH chain terminal signals.

**Table 3:** Molecular weights and thermal properties of neat PLA and the samples prepared by alcoholysis with PE and DPE as polyalcohols.

Sample code	M_n_ [g·mol^−1^]	M_w_ [g·mol^−1^]	Ð	T_g_ [°C]	T_cc_ [°C]	*Δ*H_cc_ [J·g^−1^]	T_m_ [°C]	*Δ*H_m_ [J·g^−1^]
PLA	92,000	153,000	1.66	60	−	−	−	−
PLA_PE_1	31,400	55,300	1.76	57	127	3	151	4
PLA_PE_10	24,400	35,100	1.43	55	103	36	143	34
PLA_PE_1_Zn	25,500	34,400	1.35	51	−	−	−	−
PLA_PE_10_Zn	1700	2600	1.51	21	−	−	−	−
PLA_DPE_1	48,500	80,100	1.65	59	131	4	154	5
PLA_DPE_10	40,000	62,500	1.56	56	115	34	148	34
PLA_DPE_1_Zn	41,000	53,000	1.29	54	−	−	−	−
PLA_DPE_10_Zn	5100	6600	1.29	28	−	−	−	−

M_n_ = number average molecular weight, M_w_ = weight average molecular weight, Ð = dispersity index T_g_ = glass transition temperature, T_m_ = melting temperature, ΔH_m_ = melting enthalpy, T_cc_ = cold crystallization temperature, ΔH_cc_ = cold crystallization enthalpy, T_c_ = crystallization temperature, ΔH_c_ = crystallization enthalpy.

**Table 4:** Properties of the neat polymers, GE-35H and the developed formulations based on GE-35H and the polymers from alcoholysis.

Sample code	T_g_ [°C]	T_onset_ [° C]	T_max_ [°C]	E [KPa]	σ_max_ [KPa]	ε_break_ [%]
PLA_PE_10_Zn	21	252	313	−	−	−
PLA_DPE_10_Zn	28	248	309	−	−	−
GE-35H	− 28	347	354	−	−	−
PLA_PE_10_Zn/GE-35H	−9	319	379	256 ± 3.0	29.5 ± 3.0	11.4 ± 1.6
PLA_DPE_10_Zn/GE-35H	− 25	337	393	281 ± 21	26.7 ± 8.0	11.5 ± 3.0

E = Young’s modulus; σ_max_ = tensile strength; ε_break_ = elongation at break.

## Data Availability

Data will be made available on request.
