# Supplementary material for: A sustainable approach to recycling of polylactic acid with environmentally friendly reagents
Source: Sustain Mater Technol. Author manuscript; Available in PMC 2026 Aug 7. (PMC7619334; doi:10.1016/j.susmat.2025.e01320)
Supplement: Supplementary file 1 [file EMS215456-supplement-ESI.docx]

**A sustainable approach to recycling of polylactic acid with environmentally friendly reagents**

*Giacomo Damonte^1^, Alberto Vallin^1^, Leonardo Giribaldi^1^, Alessandro Pellis^1^, Minna Hakkarainen^2^, Sathiyaraj Subramaniyan^2^, Pietro Campaner^3^, Orietta Monticelli^1*^*

*^1^Universitá degli Studi di Genova, Dipartimento di Chimica e Chimica Industriale, Via Dodecaneso 31, 16146 Genova, Italy*

*^2^KTH Royal Institute of Technology, Department of Fibre and Polymer Technology, Teknikringen 58, 100 44 Stockholm, Sweden*

*^3^AEP Polymers, Area Science Park Ed. Q1, Basovizza SS 14, km 163,5, 34149 Trieste, Italy*

**Figure S1**. FTIR of: PLA_PE_10 (black), washed PLA_PE_10 (red).

**Figure S2**. FTIR of: PLA_PE_10_Zn (black), washed PLA_PE_10_Zn (red).

**Figure S3.** Example of possible structures of star-shaped PLA oligomers obtained by alcoholysis reaction of PLA by using PE (a) or DPE (b) and ^1^H-NMR spectra of samples prepared by ROP and alcoholysis using PE (c) or DPE (d): PLA_ROP_PE (green) and PLA_PE_10_Zn (red); PLA_ROP_DPE (green) and PLA_DPE_10_Zn (red). The same colors are used for corresponding samples in both graphs.

**Figure S4.** ^1^H-NMR spectra of samples prepared by alcoholysis, with and without catalyst, using PE (a) or DPE (b): PLA_PE_1 (orange), PLA_PE_10 (light blue), PLA_PE_1_Zn (green), PLA_PE_10_Zn (purple). PLA_DPE_1 (orange), PLA_DPE_10 (light blue), PLA_DPE_1_Zn (green), PLA_DPE_10_Zn (purple). The same colors are used for corresponding samples in both graphs.

**Figure S5**. GPC curves of: PLA (a), PLA_PE_1 (b), PLA_PE_10 (c), PLA_DPE_1 (d), PLA_DPE_10 (e), PLA_PE_1_Zn (f), PLA_PE_10_Zn (g) PLA_DPE_1_Zn (h), PLA_DPE_10_Zn (i).

**Figure S6.** DSC cooling (a) and second heating (b) traces of: PLA (pink), PLA_PE_1 (green), PLA_PE_10 (dark green), PLA_DPE_1 (purple), PLA_DPE_10 (dark purple), PLA_PE_1_Zn (orange), PLA_PE_10_Zn (red) PLA_DPE_1_Zn (light blue), PLA_DPE_10_Zn (blue). The same colors are used for corresponding samples in both graphs.

**Figure S7**. Photos of: GE-35H (a), PLA_PE_10_Zn (b), the formulation after mixing at room temperature (c) and for 2 minutes at 140 °C (d).

(a)

(b)

**Figure S8**. Photos of: PLA_PE_10_Zn/GE-35H (a) and PLA_DPE_10_Zn/GE-35H (b).

**Figure S9**. FT-IR spectra of: GE-35H (red), PLA_ROP_PE (light blue), PLA_ROP_PE/GE-35H (orange), PLA_ROP_PE/GE-35H/Zn (violet), washed PLA_ROP_PE/GE-35H (green), washed PLA_ROP_PE/GE35H/Zn (pink). Full spectra (a) and zoom on the fingerprint region (b).

**Figure S10**. DSC second heating traces of GE-35H (pink), PLA_PE_10_Zn/GE-35H (violet) and PLA_DPE_10_Zn/GE-35H (orange).

**Figure S11**. Examples of stress-strain curves of PLA_PE_10_Zn/GE-35H (violet) and PLA_DPE_10_Zn/GE-35H (orange).

LC-MS analysis was performed using an Agilent 1100 HPLC system coupled with a Microsaic 4000 MiD Series mass spectrometer (ESI, Single Quadrupole). The chromatographic separation was achieved on a Phenomenex Luna C8 column (150 x 4.6 mm, 5 μm), with a pre-column. The mobile phase consisted of (A) H₂O + 0.1% formic acid and (B) CH₃CN + 0.1% formic acid. The elution profile included an isocratic phase for the first 5 minutes with 95% A, followed by a linear gradient from 5 to 35 minutes, where A was decreased from 95% to 0%. The flow rate was set at 0.8 mL/min, the injection volume was 5 μL, and the column temperature was maintained at 25°C. Mass spectrometric data were acquired in full scan mode (85-800 m/z) in negative ion mode. For LC-MS sample preparation, the supernatants recovered from enzymatic hydrolysis of star-shaped PLAs were centrifuged using an ultracentrifuge (Eppendorf MiniSpin^®^) at 14500 rpm for 5 minutes. After centrifugation, the clear liquid was recovered and filtered over a syringe filter (0.22 µm, Teflon). The filtered supernatants were diluted using 0.1% formic acid, according to their soluble fraction content (measured in the mass loss experiment), to a final concentration of 1.5 mg/mL. Differently, to avoid the precipitation of the oily fraction, the dilution of the supernatants derived from the hydrolysis of the formulations containing GE-35H was performed by using a 50:50 mixture of acetonitrile/formic acid 0.1%.

**Figure S12**. LC-MS analysis of the polymer sample PLA_DPE_10_Zn supernatant after 3 days of hydrolysis carried out in negative ion mode. TIC (top); mass spectra at 2.45 min, 3.28 min, 10.25 min and 13.33 min (bottom).

**Figure S13**. Photo of the supernatant recovered after 3 days of enzymatic hydrolysis of PLA_DPE_10_Zn (a) and PLA_DPE_10_Zn/GE-35H (b).

**Figure S14**. LC-MS analysis of the formulation PLA_DPE_10_Zn/GE-35H supernatant after 3 days of hydrolysis carried out in negative ion mode. TIC (top); mass spectra at 3.33 min, 12.72 min, 14.07 min, 15.25 min, 22.37 min and 25.67 min (bottom).
